# Acinic Cell Carcinoma of the Breast: A Population‐Based Clinicopathologic Study

**DOI:** 10.1002/cnr2.70357

**Published:** 2025-10-05

**Authors:** Faruk Skenderi, Giridhara Rathnaiah Babu, Una Glamoclija, Emir Veledar, Zoran Gatalica, Janez Lamovec, Semir Vranic

**Affiliations:** ^1^ Department of Pathology UniMed Clinic, Sarajevo School of Science and Technology Sarajevo Bosnia and Herzegovina; ^2^ College of Medicine, QU Health, Qatar University Doha Qatar; ^3^ Department of Biochemistry and Clinical Analysis University of Sarajevo Faculty of Pharmacy Sarajevo Bosnia and Herzegovina; ^4^ Scientific Research Unit, Bosnalijek d.d. Sarajevo Bosnia and Herzegovina; ^5^ Department of Neurology, Miller School of Medicine University of Miami Miami Florida USA; ^6^ Reference Medicine Phoenix Arizona USA; ^7^ Institute of Oncology Ljubljana Ljubljana Slovenia

**Keywords:** acinic cell carcinoma, breast cancer, outcome, special types, survival

## Abstract

**Purpose:**

Acinic cell carcinoma (ACC) of the breast is a very rare, primary salivary gland‐type breast malignancy, with ~100 reported cases in the literature. Limited information about the clinical features and outcomes of patients with ACC is available.

**Methods:**

We utilized the Surveillance, Epidemiology, and End Results (SEER) database to identify ACC patients. For comparison, we also examined a cohort of invasive breast carcinomas of no special type (NST).

**Results:**

Thirty ACC patients were identified among the more than 248 000 invasive breast carcinoma NST patients. ACCs were predominantly grade 3 carcinomas (44%) and were diagnosed at an earlier stage (67%). Hormone receptor (HR) and HER2 status data were available for only 13 patients, revealing molecular heterogeneity: HR−/HER2− (four patients), HR−/HER2+ (two patients), HR+/HER2− (four patients), and HR+/HER2+ (three patients). The median survival time for ACC patients was 19 months vs. 48 months for NST patients (*p* < 0.001). A complete‐case approach was utilized for the adjusted analyses, restricting the sample to 46 257 patients without missing data on all relevant covariates. The adjusted Kaplan–Meier analysis indicated a more pronounced decline in survival probabilities among patients with ACC compared to those with NST, with the number at risk in the ACC group diminishing to four patients by the 30‐month mark. In contrast, NST patients exhibited a more gradual decrease. In the multivariable Cox regression, which adjusted for age, TNM stage, HR/HER2, and chemotherapy, ACC histology was correlated with a 1.69‐fold increase in the hazard of death (HR: 1.69; 95% CI: 0.63–4.56), although this result was not statistically significant. Age and advanced stage continued to be strong predictors of poor survival, and the inclusion of an age–time interaction enhanced the model fit.

**Conclusion:**

Acinic cell carcinoma of the breast is a very rare primary breast malignancy. Our study indicates potentially aggressive clinical behavior in mammary ACC; however, findings must be interpreted cautiously given inherent SEER limitations, especially regarding histologic and molecular subtyping accuracy. Further centralized studies are urgently needed for the accurate characterization of this rare entity.

**Abbreviations**
ACC: acinic cell carcinoma
AUC: area under the ROC curve
CI: confidence interval
ER: estrogen receptor
HR: hormone receptor
HRs: hazard ratios
IHC: immunohistochemistry (immunohistochemical)
MGA: microglandular adenosis
NST: no special type
PR: progesterone receptor
ROC: receiver operating characteristic
SEER: Surveillance, Epidemiology, and End Results
TNBC: triple‐negative breast cancer
TNM: tumor, node, metastasis

## 1 Introduction

Breast cancer is the most common malignancy among women worldwide and the second leading cause of cancer‐related mortality in women [1, 2, 3]. It is a complex and heterogeneous disease with various morphologies, molecular genomes, and clinical features [2]. Invasive breast carcinoma of no special type (NST) is the most common subtype of breast cancer, constituting ~80% of all breast cancers. Special types, defined by > 90% of distinct and clinically relevant morphology, are a large and heterogeneous group of neoplasms, constituting the remaining ~20% of all breast cancers [2, 4]. Among special types, salivary gland‐type cancers are a distinct and rare group, encompassing several peculiar entities, including acinic cell carcinoma (ACC). Most salivary gland‐type breast cancers have a triple‐negative breast cancer (TNBC) phenotype [estrogen receptor (ER)‐negative, progesterone receptor (PR)‐negative, and HER2 negative]. Still, their clinical course may differ from TNBC NST [2, 5, 6, 7, 8].

The ACC is defined by its characteristic cytomorphology (clear and/or granular epithelial cells, some of which may have intracytoplasmic zymogen granules) [2, 8, 9]. The presence of eosinophilic granules resembling intestinal Paneth cells indicates ACC. However, ACC may exhibit various architectural growth patterns, ranging from microglandular adenosis (MGA)‐like to more solid growth patterns having areas of necrosis and poor differentiation. Interestingly, a subset of ACC may have a clonal relationship with MGA, while mixed variants may share molecular and genomic features with co‐existing invasive breast carcinomas NST [9, 10, 11, 12, 13]. The study of Beca et al. revealed no pathognomonic genomic alterations in ACC, but molecular features similar to TNBC NST [11]. The incidence of ACC in the breast is unknown, but our comprehensive literature survey completed in 2022 revealed only 68 well‐documented cases in the literature [6]. Our updated literature review (May 2024 and April 2025) revealed ~40 additional cases in the current literature [14, 15, 16, 17, 18]. Similar to other salivary gland‐type breast cancers, ACC usually exhibits a TNBC phenotype, with rare ER and PR positivity. However, the clinical course of patients with ACC appears less aggressive than that of patients with the NST subtype [2, 6].

In the current study, we explored the Surveillance, Epidemiology, and End Results (SEER) Program to analyze the clinicopathologic characteristics of ACC patients and compare them with those of invasive breast carcinoma NST patients.

## 2 Materials and Methods

### 2.1 Patient Selection and Cohorts

We utilized the SEER database to select our cohorts (ACC and a control cohort consisting of invasive breast carcinoma NST patients) using SEER*Stat V.8.4.0.1. The SEER database includes demographic data, tumor characteristics (such as tumor type, histologic grade, TNM stage (AJCC), tumor size, lymph node status, and the presence or absence of distant metastases), treatment options (surgery, chemotherapy, radiotherapy), and follow‐up for vital status. The SEER database encompasses clinicopathologic data from 18 population‐based cancer registries covering approximately one‐third of the American population [19].

The SEER database utilized for this study provided staging data based on the AJCC TNM 7th edition. Although the 8th edition includes important updates, SEER datasets typically lag behind the latest AJCC editions, making the 7th edition the most consistent and comparable choice for our analyses.

The status of the ER and PR (hormonal receptors [HR]) was defined based on the results of immunohistochemical (IHC) analysis. HER2 results were based on IHC and/or in situ hybridization tests. Based on the reported immunohistochemical expression of HRs and HER2 and due to the lack of Ki‐67 status in the SEER database, we used the modified (simplified) molecular classification of the cases: HR+/HER2−, HR+/HER2+, HR−/HER2+, and HR−/HER2− [19].

For our study cohort, we selected patients who were histopathologically confirmed to have ACC between 2000 and 2018. The WHO code (ICD‐O coding 8550/3 Acinar cell carcinoma) was used to identify the ACC from the SEER database. For the control cohort, we selected patients with histopathologically confirmed invasive breast carcinoma NSTs. Only patients with complete treatment and follow‐up data were included. The studies excluded all in situ carcinomas, mixed carcinomas (e.g., ductal or any other histotype combined with ACC), and other special types of breast cancers, including salivary gland‐type breast cancers.

Given the rarity of ACC, a directly matched comparator group was not feasible. For contextual interpretation of clinical characteristics and survival outcomes, we compared the ACC cohort to a large group of patients diagnosed with invasive breast carcinoma NST within the same SEER registry period. This comparison was intended to provide a descriptive benchmark to highlight potential differences in demographic, pathologic, and outcome variables. We acknowledge that invasive breast carcinoma NST represents a heterogeneous group and does not account for the histopathologic or molecular specificity of ACC. Accordingly, no direct statistical matching or subtype stratification was performed.

### 2.2 Statistical Analysis

Data from the SEER database were analyzed to compare clinicopathologic parameters between ACC and invasive ductal carcinoma NST patients using chi‐square (*χ* ^2^) tests for age and racial distributions, with significance set at *p* < 0.05. Means and standard deviations were computed for continuous variables, while frequencies and percentages were calculated for categorical variables. Mortality differences by type were stratified by stage at diagnosis. Cox proportional hazard models were used to estimate hazard ratios (HRs) and 95% confidence intervals (CIs), adjusting for stage at diagnosis, age, year of diagnosis, lymph node status, and treatment regimen. The Kaplan–Meier methods were used to generate survival curves and compare mortality by subtype, with significance assessed via the log‐rank test. Follow‐up began on the date of diagnosis (2000) and ended on the date of death, termination of health plan membership, or December 31, 2019.

Survival analyses were performed using Cox proportional hazards models to estimate HRs and 95% confidence intervals. The multivariable Cox model was adjusted for various factors, including age, molecular subtype, receptors (ER, PR, and HER2), AJCC TNM, and chemotherapy treatment. Survival time was quantified from the date of diagnosis until death or censoring at the last follow‐up. A complete case analysis was executed for the adjusted survival model, which encompassed 46 257 patients after the exclusion of those with incomplete biomarker or staging data.

Kaplan–Meier survival curves were generated to compare survival probabilities between the ACC and NST groups, with differences assessed utilizing the log‐rank test. Adjusted survival curves were plotted employing fixed covariate profiles to visualize differences after adjusting for confounding variables. The number of patients at risk was displayed at each time point beneath the survival plots.

To evaluate the assumption of proportional hazards, we generated log(−log(survival)) plots stratified by histologic type. Furthermore, logistic regression was conducted to model the odds of mortality associated with histology (ACC vs. NST), accounting for age, race, stage, grade, surgery, radiotherapy, chemotherapy, and hormonal status. Model diagnostics included a receiver operating characteristic (ROC) curve to assess discrimination and a link test to identify specification error. We used Stata version 18 [20] for the analysis.

## 3 Results

### 3.1 Clinicopathologic Characteristics of the Acinic Cell Carcinoma Cohort

The clinicopathologic and demographic characteristics of the patients in the ACC cohort are summarized in Table 1. Comparisons of the relevant clinicopathologic and demographic variables between the ACC and invasive breast carcinoma NST cohorts are shown in Table 2.

**TABLE 1:** Clinicopathologic characteristics of the patients with mammary ACC.

	Freq.	Percent
Age (levels)		
30–34 years	1	3.33
35–39 years	2	6.67
40–44 years	1	3.33
45–49 years	5	16.67
50–54 years	1	3.33
55–59 years	5	16.67
60–64 years	5	16.67
65–69 years	3	10.00
70–74 years	5	16.67
85+ years	2	6.67
Race		
Asian or Pacific Islander	2	(6.7%)
Black	2	(6.7%)
White	26	(86.7%)
Histologic grading		
Grade 1	7	(23.3%)
Grade 2	7	(23.3%)
Grade 3	11	(36.7%)
Unknown	5	(16.7%)
pT stage		
T1a	2	(6.7%)
T1b	3	(10.0%)
T1c	6	(20.0%)
T2	9	(30.0%)
T3	1	(3.3%)
pTx	7	(23.3%)
Any T, Mets	2	(6.7%)
pN stage		
N0	16	(53.3%)
N1	4	(13.3%)
N2	2	(6.7%)
N3	2	(6.7%)
NX	6	(20%)
pM stage		
M0	23	(76.7%)
M1	2	(6.7%)
MX	5	(16.6%)
AJCC TNM stage		
I	7	(23.3%)
II	13	(43.3%)
III	2	(6.6%)
IV	2	(6.7%)
Unknown	7	(20%)
Radiotherapy		
No radiotherapy	20	(66.7%)
Adjuvant radiotherapy	9	(30.0%)
The sequence is unknown, but both were given	1	(3.3%)
Surgery		
Not performed	4	(13.3%)
Surgery performed	26	(86.7%)
Chemotherapy		
Yes	14	(46.7%)
No/unknown	16	(53.3%)
Molecular subtype		
HR−/HER2−	4	(13.3%)
HR−/HER2+	2	(6.7%)
HR+/HER2−	4	(13.3%)
HR+/HER2+	3	(10.0%)
Recode not available	14	(46.7%)
Unknown	3	(10.0%)
Estrogen receptor (ER)		
Borderline/unknown	5	(16.7%)
Negative	10	(33.3%)
Positive	15	(50.0%)
Progesterone receptor (PR)		
Borderline/unknown	5	(16.7%)
Negative	10	(33.3%)
Positive	15	(50.0%)
HER‐2/neu receptor		
Borderline/unknown	3	(10.0%)
Negative	8	(26.7%)
Positive	5	(16.7%)
Recode not available	14	(46.7%)
Clinical outcome (cause of death)		
Alive or dead of another cause	23	(76.7%)
Dead (attributable to this cancer)	6	(20.0%)
Dead (missing/unknown cause of death)	1	(3.3%)

**TABLE 2:** Comparative analysis of clinicopathologic parameters between ACC and invasive ductal carcinoma NST cohorts.

Variable	ACC (*n* = 30)	NST carcinoma (*n* = 248 076)	*p*
Age			
15–19 years	0 (0.0%)	14 (< 1%)	0.63
20–24 years	0 (0.0%)	189 (0.1%)	
25–29 years	0 (0.0%)	1338 (0.5%)	
30–34 years	1 (3.3%)	3861 (1.6%)	
35–39 years	2 (6.7%)	7824 (3.2%)	
40–44 years	1 (3.3%)	14 464 (5.8%)	
45–49 years	5 (16.7%)	21 875 (8.8%)	
50–54 years	1 (3.3%)	26 400 (10.6%)	
55–59 years	5 (16.7%)	30 541 (12.3%)	
60–64 years	5 (16.7%)	34 193 (13.8%)	
65–69 years	3 (10.0%)	35 114 (14.2%)	
70–74 years	5 (16.7%)	29 308 (11.8%)	
75–79 years	0 (0.0%)	19 392 (7.8%)	
80–84 years	0 (0.0%)	12 550 (5.1%)	
85+ years	2 (6.7%)	11 013 (4.4%)	
Race			
American Indian/Alaska Native	0 (0.0%)	1685 (0.7%)	0.76
Asian or Pacific Islander	2 (6.7%)	26 005 (10.5%)	
Black	2 (6.7%)	27 423 (11.1%)	
White	26 (86.7%)	190 217 (76.7%)	
Unknown	0 (0.0%)	2746 (1.1%)	
Histologic grading			
Grade 1	7 (23.3%)	29 098 (11.7%)	0.006
Grade 2	7 (23.3%)	57 578 (23.2%)	
Grade 3	11 (36.7%)	46 110 (18.6%)	
Grade 4 (anaplastic/undifferentiated)	0 (0.0%)	34 (< 1%)	
Unknown	5 (16.7%)	115 256 (46.5%)	
pT stage			
T0	0 (0.0%)	43 (< 1%)	< 0.001
T1a	2 (6.7%)	3273 (1.3%)	
T1b	3 (10.0%)	7972 (3.2%)	
T1c	6 (20.0%)	14 915 (6.0%)	
T1mic	0 (0.0%)	949 (0.4%)	
T2	9 (30.0%)	12 482 (5.0%)	
T3	1 (3.3%)	1977 (0.8%)	
T4a	0 (0.0%)	145 (0.1%)	
T4b	0 (0.0%)	640 (0.3%)	
T4c	0 (0.0%)	22 (< 1%)	
T4d	0 (0.0%)	309 (0.1%)	
Tis	0 (0.0%)	1 (< 1%)	
TX Adjusted	3 (10.0%)	1488 (0.6%)	
Any T, Mets	2 (6.7%)	2015 (0.8%)	
Blank(s)	4 (13.3%)	201 845 (81.4%)	
pN stage			
N0	16 (53.3%)	30 820 (12.4%)	< 0.001
N1	4 (13.3%)	10 341 (4.2%)	
N2	2 (6.7%)	1894 (0.8%)	
N3	2 (6.7%)	1585 (0.6%)	
NX adjusted	2 (6.7%)	1591 (0.6%)	
Blank(s)	4 (13.3%)	201 845 (81.4%)	
pM stage			
M0	23 (76.7%)	43 593 (17.6%)	< 0.001
M1	2 (6.7%)	2015 (0.8%)	
MX	1 (3.3%)	623 (0.3%)	
Blank(s)	4 (13.3%)	201 845 (81.4%)	
AJCC TNM stage			
0	0 (0.0%)	1 (< 1%)	< 0.001
I	7 (23.3%)	22 746 (9.2%)	
IIA	13 (43.3%)	10 677 (4.3%)	
IIB	0 (0.0%)	4967 (2.0%)	
IIIA	0 (0.0%)	2429 (1.0%)	
IIIB	0 (0.0%)	884 (0.4%)	
IIIC	1 (3.3%)	1020 (0.4%)	
IIINOS	1 (3.3%)	46 (< 1%)	
IV	2 (6.7%)	2015 (0.8%)	
UNK Stage	2 (6.7%)	1446 (0.6%)	
Blank(s)	4 (13.3%)	201 845 (81.4%)	
Radiotherapy			
Intraoperative radiotherapy with other radiotherapy before/after surgery	0 (0.0%)	436 (0.2%)	< 0.001
Intraoperative radiotherapy	0 (0.0%)	2033 (0.8%)	
No radiotherapy	20 (66.7%)	122 448 (49.4%)	
Adjuvant radiotherapy	9 (30.0%)	122 125 (49.2%)	
Neoadjuvant and adjuvant radiotherapy	0 (0.0%)	388 (0.2%)	
Neoadjuvant radiotherapy	0 (0.0%)	488 (0.2%)	
The sequence is unknown, but both were given	1 (3.3%)	60 (< 1%)	
Surgery			
Surgery both before and after radiation	0 (0.0%)	98 (< 1%)	
Not performed, a patient died before the recommended surgery	0 (0.0%)	260 (0.1%)	0.94
Not performed	4 (13.3%)	18 309 (7.4%)	
Not recommended, contraindicated due to other conditions; autopsy only (1973–2002)	0 (0.0%)	1324 (0.5%)	
Recommended but not performed, patient refused	0 (0.0%)	2232 (0.9%)	
Recommended but not performed, unknown reason	0 (0.0%)	835 (0.3%)	
Recommended, unknown if performed	0 (0.0%)	2224 (0.9%)	
Surgery performed	26 (86.7%)	222 388 (89.6%)	
Unknown; death certificate; or autopsy only (2003+)	0 (0.0%)	504 (0.2%)	
Chemotherapy			
Yes	14 (46.7%)	99 123 (40.0%)	0.45
No/unknown	16 (53.3%)	148 953 (60.0%)	
Molecular subtype			
HR−/HER2−	4 (13.3%)	27 864 (11.2%)	< 0.001
HR−/HER2+	2 (6.7%)	11 748 (4.7%)	
HR+/HER2−	4 (13.3%)	168 077 (67.8%)	
HR+/HER2+	3 (10.0%)	28 179 (11.4%)	
Recode not available	14 (46.7%)	0 (0.0%)	
Unknown	3 (10.0%)	12 208 (4.9%)	
Estrogen receptor (ER)			
Borderline/unknown	5 (16.7%)	4699 (1.9%)	< 0.001
Negative	10 (33.3%)	43 556 (17.6%)	
Positive	15 (50.0%)	199 821 (80.5%)	
Progesterone receptor (PR)			
Borderline/unknown	5 (16.7%)	5093 (2.1%)	< 0.001
Negative	10 (33.3%)	68 487 (27.6%)	
Positive	15 (50.0%)	174 496 (70.3%)	
HER‐2/neu receptor			
Borderline/unknown	3 (10.0%)	11 884 (4.8%)	< 0.001
Negative	8 (26.7%)	196 159 (79.1%)	
Positive	5 (16.7%)	40 033 (16.1%)	
Recode not available	14 (46.7%)	0 (0.0%)	
Clinical outcome (cause of death)			
Alive or dead of another cause	23 (76.7%)	231 137 (93.2%)	
Dead (attributable to this cancer)	6 (20.0%)	16 461 (6.6%)	< 0.001
Dead (missing/unknown cause of death)	1 (3.3%)	463 (0.2%)	

Thirty ACC cases and approximately 248 000 invasive breast carcinoma NST cases were retrieved from 2000 to 2018, indicating that the frequency of ACC was approximately 0.01%. All ACC patients were women, with no cases found in men. Most ACC patients (19/30) were between 45 and 69 years old. There was no significant difference in age between ACC patients and those with invasive breast carcinoma NSTs (Table 2, *p* = 0.63). Similar to invasive breast carcinoma NSTs, ACC predominantly affects the white population (26/30). As expected, the two cohorts differed in most clinicopathologic variables, including histologic grade, tumor size (pT), axillary lymph node status (pN), and the presence or absence of distant metastases (pM) (Table 2).

Nottingham histologic grading was available for 25 cases, out of which 11 (44%) were high‐grade (G3) carcinomas. Two‐thirds of the patients presented with early‐stage breast cancers (pT1–2 and N0). Surgery was the primary treatment modality for 86.7% of ACC patients, while adjuvant radiotherapy and chemotherapy were administered to 9/29 and 14/30 patients, respectively (Table 1).

### 3.2 Molecular Characteristics of the Acinic Cell Carcinoma Cohort

The status of HR and HER2 receptors, which are used as surrogates for molecular classification in the ACC cohort, was available for 13 patients. Four patients were HR−/HER2−, two were HR−/HER2+−, and seven had a luminal phenotype. Four of the luminal cases were HR+/HER2−, and three were luminal HR+/HER2+ ACC (HR+/HER2+) (Table 1).

### 3.3 Survival and Outcomes of ACC Patients

After removing incomplete cases, the final adjusted analysis included 46 257 patients, of whom 26 were diagnosed with ACC and the rest with invasive breast carcinoma NST. The median survival for patients with ACC was 19 months and 48 months for those with NST. The average follow‐up duration was 21.4 months for the ACC group and 52.2 months for NST.

**FIGURE 1:**
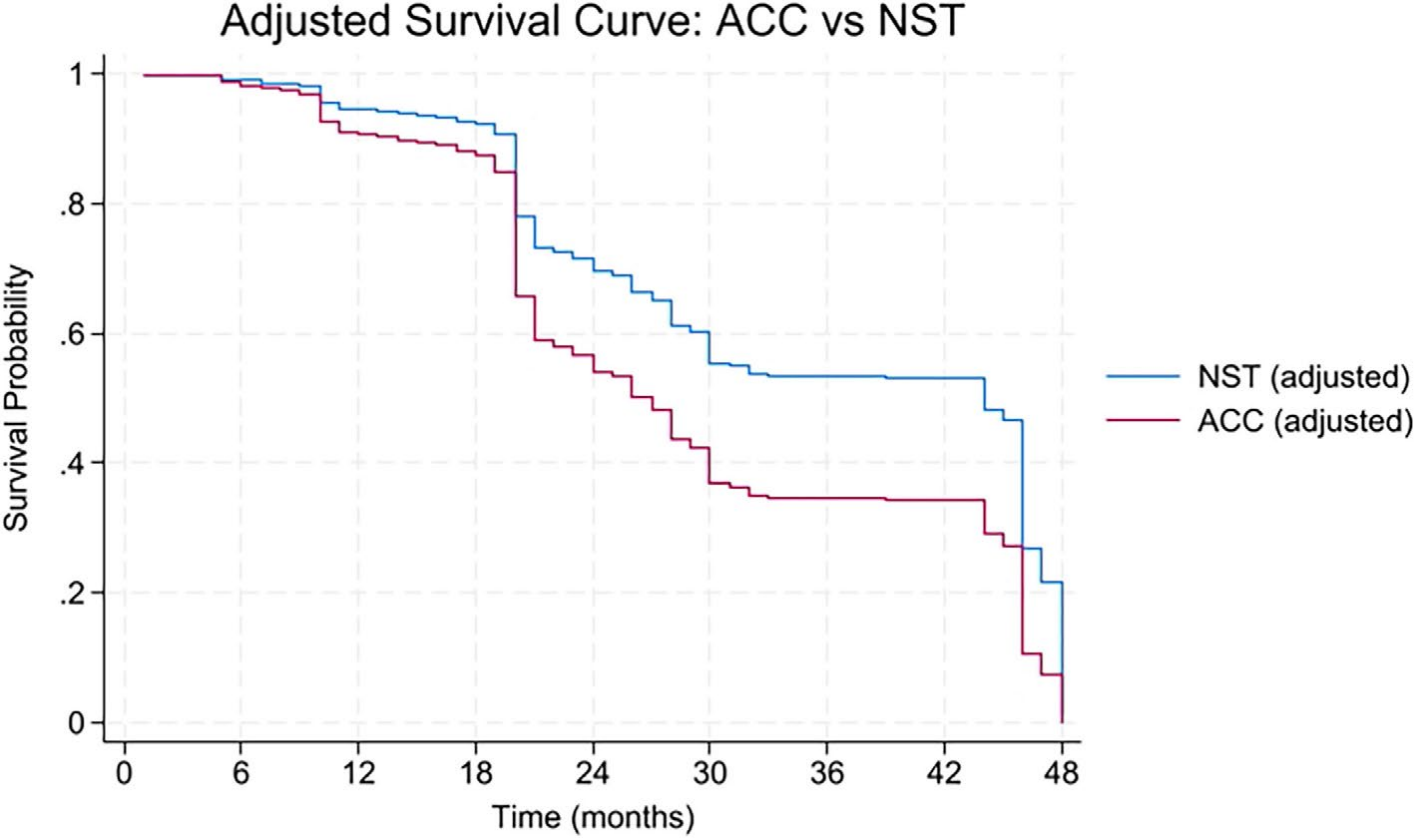
Kaplan–Meier survival analysis demonstrated significantly lower survival probabilities for ACC patients than NST (log‐rank *p* = 0.0006). The accompanying risk table showed a steep decline in the number of at‐risk ACC patients over time, particularly after 24 months, whereas NST patients declined more gradually.

The Kaplan–Meier analysis (Figure 1) indicated a significantly lower survival rate in patients diagnosed with ACC compared to those with invasive breast carcinoma NST (log‐rank *p* = 0.0006). The decline was steep in survival rates for ACC patients occurring after 24 months. In contrast, NST cases displayed a more gradual decrease in survival over time. The unadjusted survival curve distinctly illustrated a survival disadvantage associated with ACC (not shown).

After adjusting for age, molecular subtype, ER/PR/HER2 status, AJCC TNM, and chemotherapy, the Cox proportional hazards model indicated that ACC was associated with a 1.69‐fold increased hazard of death (HR = 1.69; 95% CI: 0.63–4.56) (Table 3); however, this finding did not find statistical significance (*p* = 0.296). Age emerged as a strong independent predictor of mortality (HR = 1.70; *p* < 0.001), as did higher AJCC stages. Specifically, AJCC stages III and IV demonstrated a 1.83‐fold and a 2.49‐fold increased hazard, respectively (*p* < 0.001). Subtypes 2–4 (HER2+) exhibited significant protective effects, whereas the ER/PR/HER2 status did not yield significant associations in this model. Furthermore, chemotherapy displayed a modest but statistically significant association with an increased hazard (HR = 1.07; *p* = 0.028) (Table 3).

**TABLE 3:** Cox proportional hazards regression^#^ of ACC patients vs. patients with invasive ductal carcinoma NST.

_t	Coef.	St. Error.	*t*‐value	*p*	[95% Conf Interval]		Sig
Case	1.695	0.855	1.05	0.296	0.63	4.556	
Age	1.7	0.013	69.60	0	1.675	1.726	***
Subt: base 1	1	.	.	.	.	.	
2	0.65	0.033	−8.39	0	0.588	0.719	***
3	0.535	0.017	−20.11	0	0.504	0.569	***
4	0.548	0.022	−14.80	0	0.506	0.594	***
5	0.601	0.415	−0.74	0.46	0.156	2.323	
6	0.772	0.227	−0.88	0.377	0.434	1.372	
ER_bin	1.268	0.411	0.73	0.465	0.671	2.393	
PR_bin	0.802	0.257	−0.69	0.49	0.428	1.502	
HER2_bin	0.763	0.22	−0.94	0.349	0.434	1.344	
ajcc15: base 1	1	.	.	.	.	.	
2	1.468	0.039	14.30	0	1.393	1.547	***
3	1.834	0.081	13.74	0	1.682	2	***
4	2.491	0.107	21.16	0	2.289	2.71	***
5	1.167	0.054	3.31	0.001	1.065	1.278	***
Chemo	1.067	0.032	2.20	0.028	1.007	1.131	
Age_ln_time	0.828	0.002	−93.77	0	0.825	0.831	***

Mean dependent var	8.581	SD dependent var.	7.247
Pseudo *R*‐squared	0.102	Number of obs	46 257
Chi‐square	9595.747	Prob > chi^2^	0.000
Akaike crit. (AIC)	144199.906	Bayesian crit. (BIC)	144339.778

*** *p* < 0.01.

The Schoenfeld residuals were used to assess the assumption of proportional hazards. The global test revealed a violation of proportionality (*p* < 0.001), predominantly influenced by age and advanced AJCC stages. An interaction term between age and log(time) (“age_lntime”) was incorporated to account for time‐varying effects. This adjustment enhanced model fit, resolved proportionality concerns, and preserved the interpretability of the coefficients.

The plot of deviance residuals by age (Figure S1) revealed no significant patterns or heteroskedasticity, indicating a suitable model specification. The log–log survival plot (Figure S2) exhibited slight non‐parallelism between ACC and NST early in the follow‐up period, which is consistent with the initial Schoenfeld diagnostics and justifies the inclusion of time‐dependent effects.

## 4 Discussion

Salivary gland‐type tumors of the breast are rare primary mammary malignancies consisting of six well‐defined entities that typically exhibit triple‐negative phenotypes and have clinically intermediate aggressive potential [2]. As confirmed by our study, mammary ACC is exceedingly rare, comprising only 30 cases out of more than 248 000 invasive breast carcinoma NST cases analyzed. Despite similar treatment options, our data indicate that patients with mammary ACC may have worse outcomes than those with the more common NST subtype of breast carcinoma. Despite earlier clinical presentations (pT1–2No stage in 2/3 of the cohort), ACC patients had substantially shorter median survival times than IDC NST patients, with a 1.69‐fold greater risk of death, indicating a potentially worse prognosis. Despite their heterogeneous molecular profile and subtypes, our study revealed that ACC may have a more aggressive clinical course, resembling ER‐negative breast cancers [21]. The adjusted survival rates for ACC patients revealed a decline in the number of at‐risk patients over time, beginning at approximately 2 years and dropping quickly. In contrast, the IDC NST group experienced a more gradual decrease. This pattern aligns with the recurrence trend observed in patients with ER‐negative breast cancers, in which recurrence risk peaks ~3 years postdiagnosis and then swiftly decreases [21].

One‐third of ACC patients in our cohort presented with axillary lymph node metastases, partially explaining the more aggressive clinical course and poor outcomes of ACC patients. These findings contrast with previous data showing a low frequency of axillary lymph node metastases in patients with mammary ACC [2, 6, 22, 23]. For instance, Zhong et al. reported no axillary metastases among 11 ACC patients in their cohort [24]. On the other hand, Guerini‐Rocco et al. in a cohort of seven acinic cell carcinomas (two pure and five mixed cases) reported axillary lymph node metastases in two cases, of which one was pure ACC. They also reported the disease recurrence or ACC‐related death in three out of five patients with available follow‐up [10]. Kim et al. also noted axillary lymph node metastases in one out of three ACC cases from their cohort [25] (Supplemental File 1).

Another significant observation from the SEER database is the molecular heterogeneity among ACC patients. Although HR and HER2 status data were available for only 13 patients, approximately half were ER‐negative, and only four out of 13 patients were TNBC. This finding contrasts with most previous studies, which indicated a predominantly triple‐negative phenotype, with approximately 10% of ACC patients being positive for ER and PR, and no HER2 positivity reported [2, 6]. The only study reporting HER2 expression in mammary ACC is a recent publication by Ch'ng ES [26], which also examined the SEER database for a different salivary gland‐type breast cohort, including a subset of ACC patients. Given the nature of the SEER database, re‐reviewing previously diagnosed ACC records is not possible. However, due to the inconsistent results obtained from the SEER database, it is reasonable to believe that a certain number of ACC cases do not represent pure ACC but mixed (ductal NST and ACC) carcinomas or may represent other histologic subtypes of breast cancer (e.g., metaplastic, secretory, or some other salivary gland‐type cancers) [14, 25]. In addition, the diverse morphologic appearance of ACC may be another source of confusion and a potential misdiagnosis [14]. Therefore, our results should be cautiously interpreted due to a potential misclassification (misdiagnosis) of mammary ACC.

Our data show a predominance of ACC among individuals of Caucasian descent. While TNBCs are more prevalent among Black and Asian populations, ACC is a histologically and molecularly distinct subtype of TNBC. The observed racial distribution in ACC may reflect underlying genetic or epigenetic predispositions that differ from those driving conventional TNBC. However, due to the rarity of ACC and the limited number of cases reported in the study, conclusive epidemiologic patterns remain difficult to establish. It is also possible that detection and referral biases in predominantly Caucasian regions or institutions contribute to this observation. Further population‐based studies with broader ethnic representation are warranted to clarify whether this trend is consistent and biologically grounded.

The SEER database has several inherent limitations. These include incomplete information for a significant proportion of cases, particularly regarding clinically relevant variables such as tumor grade, stage, and the status of HR and HER2. The histopathologic diagnosis of ACC may be uncertain in many cases, particularly in light of the discrepancies observed in molecular profiling (HR and HER2) between our findings and those reported in the existing literature [6, 17]. Additionally, detailed information on specific treatment modalities and the duration of their use is often missing. Consequently, the survival data in SEER may be influenced by variations in treatment practices, diagnostic accuracy, and coding consistency. Another important limitation of our analysis lies in the comparison between ACC and invasive breast carcinoma NST. While the two groups differ significantly in frequency and biological profile, using NST as a reference group was not intended to imply equivalency but rather to provide a broader clinical context for understanding ACC's presentation and outcomes. We fully acknowledge the potential for bias due to a lack of histologic stratification in the comparator group. However, in the absence of robust, population‐level datasets for rare breast cancer subtypes, this approach still offers a valuable preliminary epidemiologic overview. Future research using molecularly annotated or centrally reviewed datasets is warranted to further elucidate ACC's clinical behavior. In addition, recent literature underscores ACC's molecular heterogeneity, highlighting potential overlap with MGA and mixed high‐grade carcinomas. Our study, constrained by SEER's limitations, reinforces the necessity of comprehensive histologic and molecular characterization for accurate clinical and prognostic interpretations.

In conclusion, our population‐based cohort study of ACC of the breast confirms its rarity. Our study also highlights potentially aggressive clinical behavior in mammary ACC; however, findings must be interpreted cautiously given inherent SEER limitations, especially regarding histologic and molecular subtyping accuracy. Further centralized studies are urgently needed for the accurate characterization of this rare entity.

## Author Contributions

Conceptualization: Faruk Skenderi and Semir Vranic. Data curation: Faruk Skenderi, Giridhara Rathnaiah Babu, Una Glamoclija, Emir Veledar, and Semir Vranic. Formal analysis: Faruk Skenderi, Giridhara Rathnaiah Babu, Una Glamoclija, Emir Veledar, Zoran Gatalica, Janez Lamovec, and Semir Vranic. Funding acquisition: Semir Vranic. Investigation: Faruk Skenderi, Giridhara Rathnaiah Babu, Una Glamoclija, Emir Veledar, Zoran Gatalica, Janez Lamovec, and Semir Vranic. Methodology: Faruk Skenderi, Giridhara Rathnaiah Babu, Una Glamoclija, Emir Veledar, and Semir Vranic. Software: Giridhara Rathnaiah Babu, Una Glamoclija, and Emir Veledar. Supervision: Semir Vranic. Roles/Writing – original draft: Faruk Skenderi, Giridhara Rathnaiah Babu, and Semir Vranic. Writing – review and editing: Faruk Skenderi, Giridhara Rathnaiah Babu, and Semir Vranic.

## Ethics Statement

Ethical approval was not required, given that the SEER database is publicly available with fully anonymized patient data. The corresponding author also signed a SEER Research Data Agreement to use the data for research purposes (SEER ID: 17257‐Nov2018, signed on October 24, 2019).

## Conflicts of Interest

Una Glamoclija is an employee of Bosnalijek d.d., Sarajevo, Bosnia and Herzegovina. The other authors have no relevant financial or nonfinancial interests to disclose.

## Supporting information


**Supplemental File 1.** Comparisons between the SEER cohort and another two previously published cohorts (Guerini‐Rocco et al. 2015 and Kim et al. 2017).

**Figure S1:** Deviance residuals plotted against age revealed no substantial heteroskedasticity or non‐linearity, supporting the adequacy of the model specification.

**Figure S2:** Log–log survival curves confirmed non‐parallelism between ACC and NST groups early in follow‐up, consistent with the proportional hazards violation initially observed.

## Data Availability

The datasets from the study can be obtained from the corresponding author upon reasonable request.
