# Supplementary material for: Acinic Cell Carcinoma of the Breast: A Population‐Based Clinicopathologic Study
Source: Cancer Rep (Hoboken). 2025 Oct 5;8(10):e70357. doi: 10.1002/cnr2.70357 (PMC12496478; doi:10.1002/cnr2.70357)
Supplement: Supplementary file 1 — Supplemental File 1. Comparisons between the SEER cohort and another two previously published cohorts (Guerini‐Rocco et al. 2015 and Kim et al. 2017). [file CNR2-8-e70357-s002.docx]

**Supplemental File 1: Comparison between SEER data and two key publications (Guerini-Rocco et al. 2015 and Kim et al. 2017).**

| Study | Sample Size | Median survival (months) |
| --- | --- | --- |
| SEER Data | 30 | ~19 |
| Guerini-Rocco et al. 2015 | 8 | Not reached (long-term survivors) |
| Kim et al. 2017 | 5 | N/A (only 1 recurrence) |

| **Study** | **TP53 Mutation** | **BRCA1 Mutation** | **Subtype** | **Ki67 (%)** | **Recurrence Rate** |
| --- | --- | --- | --- | --- | --- |
| SEER Data | Unknown | Unknown | Mixed (likely includes aggressive forms) | Unknown | Not reported (20% cancer-specific mortality) |
| Guerini-Rocco et al. 2025 | 7/8 cases | 2/8 (1 somatic, 1 germline) | 2 Pure, 6 Mixed | Low to Intermediate | Not reported; presumed low |
| Kim et al. 2017 | 1/5 (p53 IHC+) | Not tested | 3 with acinic features from MGA | 5  80% (case dependent) | 1/5 (20%) |

**SEER Data: Median OS: ~19, TP53: Unknown, BRCA1: Unknown, Subtype: Mixed (likely includes aggressive forms), Ki67: Unknown, Recurrence: not reported (20% cancer-specific mortality), Treatment: Registry date – unknown**

**Guerini-Rocco et al. 2015: Median OS: Not reached (long-term survivors), TP53: 7/8 cases, BRCA1: 2/8 (1 somatic, 1 germline), Subtype: 2 Pure, 6 Mixed, Ki67: Low to Intermediate, Recurrence: Not reported; presumed low, Treatment: Not specified (retrospective)**

**Kim et al. 2017: Median OS: N/A (only 1 recurrence), TP53: 1/5 (p53 IHC+), BRCA1: Not tested, Subtype: 3 with acinic features from MGA, Ki67: 5–80% (case dependent), Recurrence: 1/5 (20%), Treatment: Breast-conserving + CRT**
