# Supplementary figures and images for: Acinic Cell Carcinoma of the Breast: A Population‐Based Clinicopathologic Study
Source: Cancer Rep (Hoboken). 2025 Oct 5;8(10):e70357. doi: 10.1002/cnr2.70357 (PMC12496478; doi:10.1002/cnr2.70357)

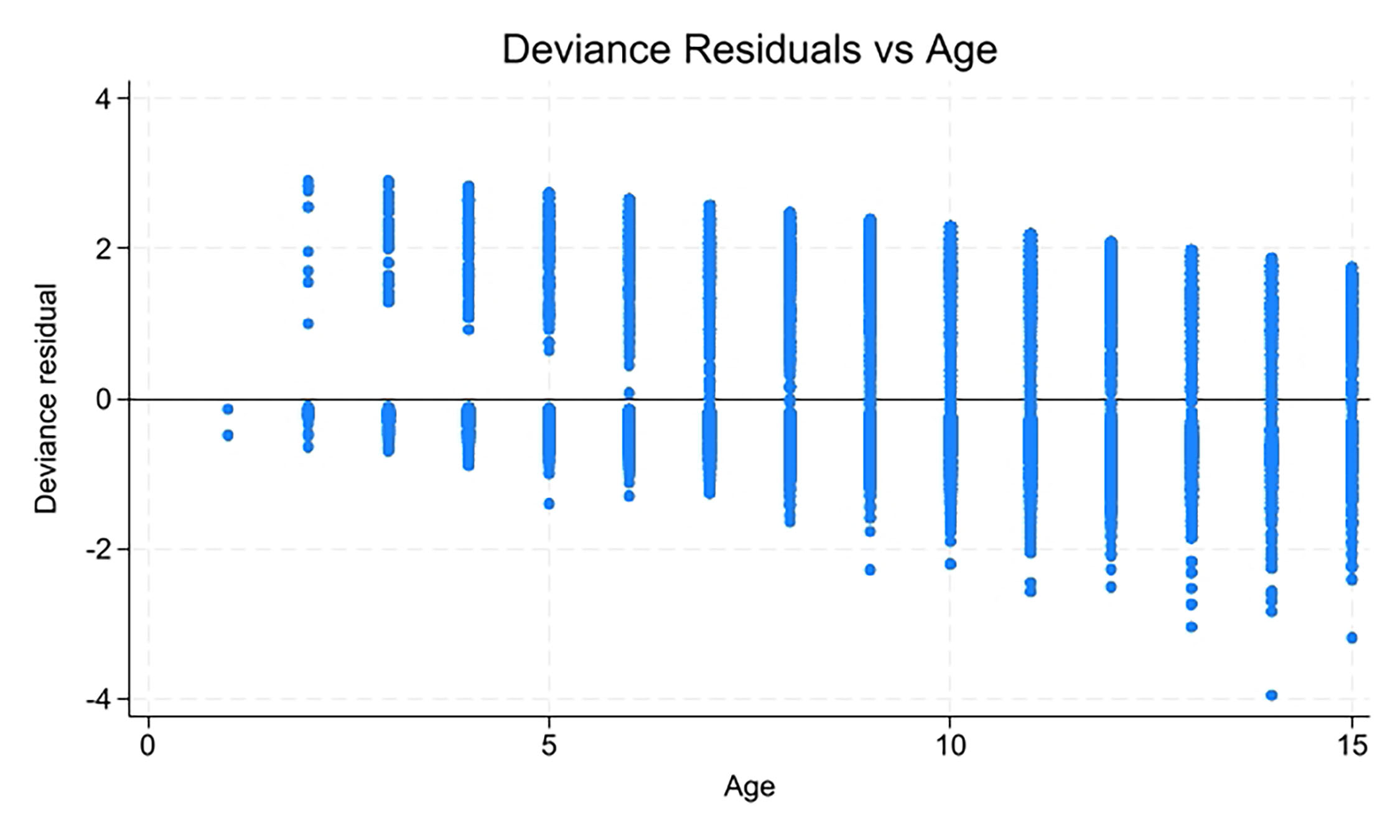

Supplement: Supplementary file 2 — Figure S1: Deviance residuals plotted against age revealed no substantial heteroskedasticity or non‐linearity, supporting the adequacy of the model specification. [file CNR2-8-e70357-s003.jpg]

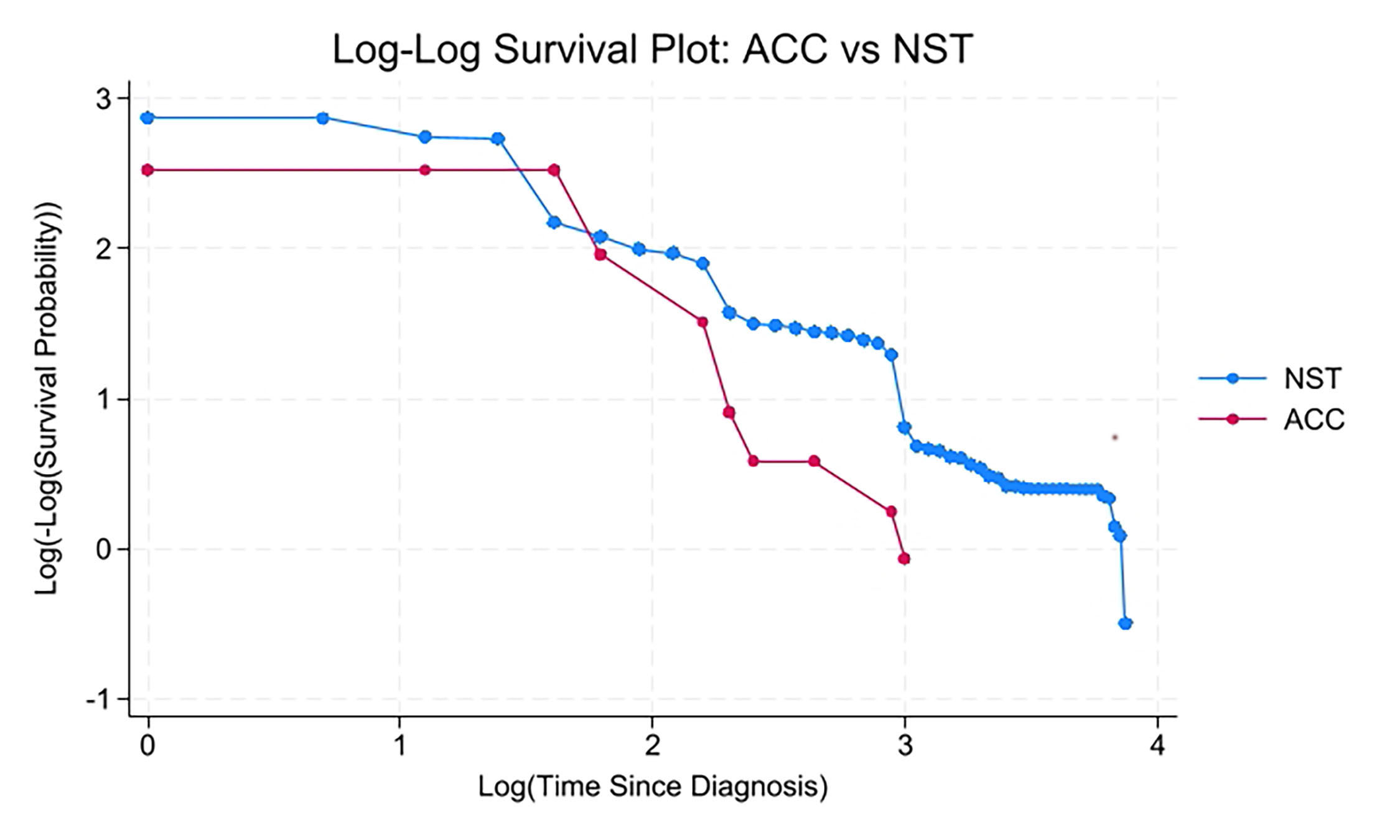

Supplement: Supplementary file 3 — Figure S2: Log–log survival curves confirmed non‐parallelism between ACC and NST groups early in follow‐up, consistent with the proportional hazards violation initially observed. [file CNR2-8-e70357-s001.jpg]
